# Ozone: Kinney et al. Respond

**Published:** 2005-02

**Authors:** Patrick L. Kinney, Kim Knowlton, Christian Hogrefe

**Affiliations:** Department of Environmental Health Sciences, Mailman School of Public Health and Columbia, University E-mail: plk3@columbia.edu; State University of New York at Albany, Albany, New York

We appreciate the interest taken by Schwartz et al. in our recent article (Knowlton et al. 2004) and welcome the opportunity to respond to their comments. Although they raised several important issues regarding ozone trends, we do not believe these issues are as relevant to our article as they suggest. Further, we wish to highlight several points on which their letter is misleading.

The overall aim of our article was to illustrate the use of a new modeling system that could be of value to policy makers engaged in projecting future ozone levels and corresponding health impacts. Our contention is that, because ozone levels are so sensitive to climate, analyses of future ozone impacts ought to take climate change into account. The overall thrust of the article was summarized in the concluding sentence of the abstract:

This modeling framework provides a potentially useful new tool for assessing the health risks of climate change.

Schwartz et al. suggest incorrectly that our model runs were intended to project what is likely to actually happen with ozone and mortality in the 2050s under a changing climate. Had we wished to do that, we would have needed to include realistic estimates of ozone precursor changes over the period of interest. However, because there are no reliable estimates of precursor emissions extending to the mid 21st century, such an exercise would have been extremely speculative. To avoid this bind, for our main analysis of climate impacts on ozone we chose to simply keep anthropogenic ozone precursor emission levels constant. We used 1996 U.S. Environmental Protection Agency (EPA) emissions estimates (U.S. EPA 2002) because those fell within our reference period. We do not dispute Schwartz et al.’s point that precursor emissions have likely decreased since 1996. We do note, however, that as a result of the complex chemistry of ozone formation, ambient ozone concentrations do not necessarily change in direct proportion to precursor changes (Seinfeld and Pandis 1998). These relationships become even more complex when both precursor changes and changing climate are taken into consideration (Hogrefe et al. 2004). To illustrate this complexity, when we allowed regional ozone precursor emissions to rise in a manner consistent with the Intergovernmental Panel on Climate Change (IPCC) A2 scenario (Nakicenovic and Swart 2000) in a sensitivity analysis, the ambient ozone levels projected for several urban counties around New York City, New York, in the 2050s actually decreased from 1990s levels (Knowlton et al. 2004, Table 1). As a result, Schwartz et al.’s statement that we assume ozone precursor emissions several times greater than any plausible future scenario, and as a result our projections of future ozone and related health impacts are unrealistically high, is misleading and probably incorrect.

We also wish to point out that, in spite of the recent reductions in precursor emissions that Schwartz et al. highlight, the U.S. EPA has reported that nationwide ozone levels have decreased only slightly since 1990 [U.S. EPA (2004), Figure 9]. This likely reflects the complex interplay between precursors and climate in the formation and dispersion of ozone. Therefore, it is not at all clear what impact the introduction of more realistic ozone precursor emission estimates would have on future ozone levels in the New York City region. However, addressing such questions is exactly what we hope our modeling system will be used for in the future.

Schwartz et al. highlight one sentence from the end of a discussion paragraph to imply that we downplayed the significance of air conditioning as an adaptive strategy to a warming climate. In fact, the main thrust of the paragraph was to acknowledge air conditioning as a potential tool for reducing the health impacts of both heat and ozone. The excerpted sentence was merely a cautionary reminder that our adaptive mechanisms have in the past been vulnerable to disruption at times, that is, during extreme heat waves, when they are most needed.

In conclusion, we hope that even discriminating readers will be impressed by the utility of this new modeling system to provide down-scaled estimates of general circulation models and associated air quality, and will be motivated to use this system to evaluate alternative inputs and their potential impacts on future climate and air quality at fine spatial scales in the United States.
